# MBEToolbox: a Matlab toolbox for sequence data analysis in molecular biology and evolution

**DOI:** 10.1186/1471-2105-6-64

**Published:** 2005-03-22

**Authors:** James J Cai, David K Smith, Xuhua Xia, Kwok-yung Yuen

**Affiliations:** 1 Department of Microbiology, University of Hong Kong, Pokfulam, Hong Kong, China; 2 Department of Biochemistry, University of Hong Kong, Pokfulam, Hong Kong, China; 3 Department of Biology, University of Ottawa, Canada

## Abstract

**Background:**

MATLAB is a high-performance language for technical computing, integrating computation, visualization, and programming in an easy-to-use environment. It has been widely used in many areas, such as mathematics and computation, algorithm development, data acquisition, modeling, simulation, and scientific and engineering graphics. However, few functions are freely available in MATLAB to perform the sequence data analyses specifically required for molecular biology and evolution.

**Results:**

We have developed a MATLAB toolbox, called MBEToolbox, aimed at filling this gap by offering efficient implementations of the most needed functions in molecular biology and evolution. It can be used to manipulate aligned sequences, calculate evolutionary distances, estimate synonymous and nonsynonymous substitution rates, and infer phylogenetic trees. Moreover, it provides an extensible, functional framework for users with more specialized requirements to explore and analyze aligned nucleotide or protein sequences from an evolutionary perspective. The full functions in the toolbox are accessible through the command-line for seasoned MATLAB users. A graphical user interface, that may be especially useful for non-specialist end users, is also provided.

**Conclusion:**

MBEToolbox is a useful tool that can aid in the exploration, interpretation and visualization of data in molecular biology and evolution. The software is publicly available at  and .

## Background

MATLAB integrates programming, visualization and computation in an easy-to-use environment and is widely used in scientific and engineering studies. One of the most attractive features of MATLAB is that the basic data element of the system is a matrix that does not require dimensioning. This allows users to solve many technical computing problems, especially those with matrix and vector formulations, in a very effective way. The MATLAB environment itself offers a comprehensive set of built-in functions and many toolboxes have been developed, and are often freely available, for more specialized needs.

However, to our knowledge, these advantages in the MATLAB environment have not been fully utilized in the area of molecular biology and evolution. Only a few MATLAB toolboxes or functions are freely available for data analysis, exploration, and visualization of nucleotide and protein sequences. MATHWORKS has recently provided a bioinformatics toolbox, however this toolbox has relatively limited functions for molecular evolutionary studies. MBEToolbox, is presented here to fulfil the most obvious needs in sequence manipulation, genetic distance estimation and phylogeny inference under the MATLAB environment. Moreover, this toolbox provides an extensible, functional framework to formulate and solve problems in evolutionary data analysis. It facilitates the rapid construction of both general applications, as well as special-purpose tools for evolutionary biologists, in a fraction of the time it would take to write a program in a scalar, noninteractive language such as C or FORTRAN.

## Implementation

MBEToolbox is written in the MATLAB language and has been tested on the WINDOWS platform with MATLAB version 6.1.0. The main functions implemented are: sequence manipulation, computation of evolutionary distances derived from nucleotide-, amino acid- or codon-based substitution models, phylogenetic tree construction, sequence statistics and graphics functions to visualize the results of analyses. Although it implements only a small fraction of the multiplicity of existing methods used in molecular evolutionary analyses, interested users can easily extend the toolbox.

### Input data and formats

MBEToolbox requires a single ASCII file containing the nucleotide or amino acid sequence alignment in either PHYLIP [1], CLUSTALW [2] or fasta format. The toolbox does provide a built-in CLUSTALW [2] interface if an unaligned sequence file is provided. Protein-coding DNA sequences can be automatically aligned based on the corresponding protein alignment with the command alignseqfile.

After input, in common with the MATHWORKS bioinformatics toolbox, MBEToolbox represents the alignment as a numeric matrix with every element standing for a nucleic or amino acid character. Nucleotides A, C, G and T are converted to integers 1 to 4, and the 20 amino acids are converted to integers 1 to 20. A header, containing information about the names and type of the sequences as well as the relevant genetic code for protein-coding nucleotides, is attached to the alignment matrix to form a MATLAB *structure*. An example alignment structure, aln, in MATLAB code follows:

aln =

seqtype: 2

geneticcode: 1

seqnames: {1 × n cell}

seq: [n × m double]

where *n *is the number of sequences and *m *is the length of the aligned sequences. The type of sequence is denoted by 1, 2 or 3 for sequences of non-coding nucleotides, protein coding nucleotides and amino acids, respectively.

### Sequence manipulation and statistics

The alignment structure, aln, can be manipulated using the MATLAB language. For example, aln.seq(x,:) will extract the *x*th sequence from the alignment, while aln.seq(:, [i: j]) will extract columns *i *to *j *from the alignment. Users may easily extract more specific positions by using functions developed in the toolbox, such as extractpos(aln, 3) or extractdegeneratesites to obtain the third codon positions or fourfold degenerate sites, respectively. For each sequence, some basic statistics such as the nucleotide composition (ntcomposition) and GC content, can be reported. Other functions include the calculation of the relative synonymous codon usage (RSCU) and the codon adaptation index (CAI), counts of segregating sites, taking the reverse complement or translating a sequence, and determining the sequence complexity.

### Evolutionary distances

The evolutionary distance is one of the important measures in molecular evolutionary studies. It is required to measure the diversity among sequences and to infer distance-based phylogenies. MBEToolbox contains a number of functions to calculate evolutionary distances based on the observed number of differences. The formulae used in these functions are analytical solutions of a variety of Markov substitution models, such as JC69 [3], K2P [4], F84 [1], HKY [5] (see [6] for detail). Given the stationarity condition, the most general form of Markov substitution models is the General Time Reversible (GTR or REV) model [7-10]. There is no analytical formula to calculate the GTR distance directly. A general method, described by Rodriguez et al. [9], has been implemented here. In this method a matrix **F**, where *F*_*ij *_denotes the proportion of sites for which sequence 1 (*s*_1_) has an *i *and sequence 2 (*s*_2_) has a *j*, is formed. The GTR distance between *s*_1 _and *s*_2 _is then given by



where **∏ **denotes the diagonal matrix with values of nucleotide equilibrium frequencies on the diagonal, and *tr*(**A**)denotes the trace of matrix **A**. The above formula can be expressed in MATLAB syntax directly as:

>> d = -trace(PI*logm(inv(PI)*F))

MBEToolbox also calculates the gamma distribution distance and the LogDet distance [11] (i.e., Lake's paralinear distance [12]).

For alignments of codons, the toolbox provides calculation or estimation of the synonymous (*K*_*s*_) and non-synonymous (*K*_*a*_) substitution rates by the counting method of Nei and Gojobori [13], the degenerate methods of Li, Wu and Luo [14] and the method of Li or Pamilo and Bianchi [15,16], as well as the maximum likelihood method through PAML [17]. All these methods for calculating *K*_*s *_and *K*_*a *_require that the input sequences are aligned in the appropriate reading frame, which can be performed by the function alignseqfile. Unresolved codon sites will be removed automatically. In addition, several quantities, including the number of substitutions per site at only synonymous sites, at only non-synonymous sites, at only four-fold-degenerate sites, or at only zero-fold-degenerate sites can be calculated. The output from these calculations are distance matrices which can be exported into text or excel files, or used directly in further operations.

### Phylogeny inference

Two distance-based tree creation algorithms, Unweighted Pair Group Method with Arithmetic mean (UPGMA) and neighbor-joining (NJ) [18] are provided and trees from these methods can be displayed or exported. Maximum parsimony and maximum likelihood algorithms can be applied to nucleotide or amino acid alignments through an interface to the phylip package [1]. As properly implemented maximum likelihood methods are the best vehicles for statistical inference of evolutionary relationships among species from sequence data, several maximum likelihood functions have been explicitly implemented in MBEToolbox. These functions allow users to incorporate various evolutionary models, estimate parameters and compare different evolutionary trees.

The simplest case of estimation of the evolutionary distance between two sequences, *s*1 and *s*2, can be considered as the estimation of the branch length (the number of substitutions along a branch) separating ancestor and descendent nodes. Branch lengths, relative to a calibrated molecular clock, can reveal the time interval for this separation. A continuous time Markov process is generally used to model evolution along the branch from *s*1 to *s*2. A *transition rate matrix*, **Q**, is used to indicate the rate of changing from one state to another. For a specified time interval or distance, *t*, the *transition probability matrix *is calculated from **P**(*t*) = *e*^***Q**t*^. If there are *N *sites, the full likelihood is



In this equation,  and  are the *i*th bases of sequences 1 and 2 respectively;  is the expected frequency of base .

In MBEToolbox, to calculate the likelihood, *L*, at a given time interval (or distance) *t*, we have to specify a substitution model by using an appropriate model defining function, such as modeljc, modelk2p or modelgtr for non-coding nucleotides, modeljtt or modeldayhoff for amino acids, or modelgy94 for codons. These functions return a model structure composed of an *instantaneous rate matrix*, R, and an equilibrium frequency vector, pi which give **Q**, (Q = R*diag(pi)). Once the model is specified, the function likelidist(t, model, s1, s2) can calculate the log likelihood of the alignment of the two sequences, *s*1 and s2, with respect to the time or distance, *t*, under the substitution model, *model*.

**Figure 1:**
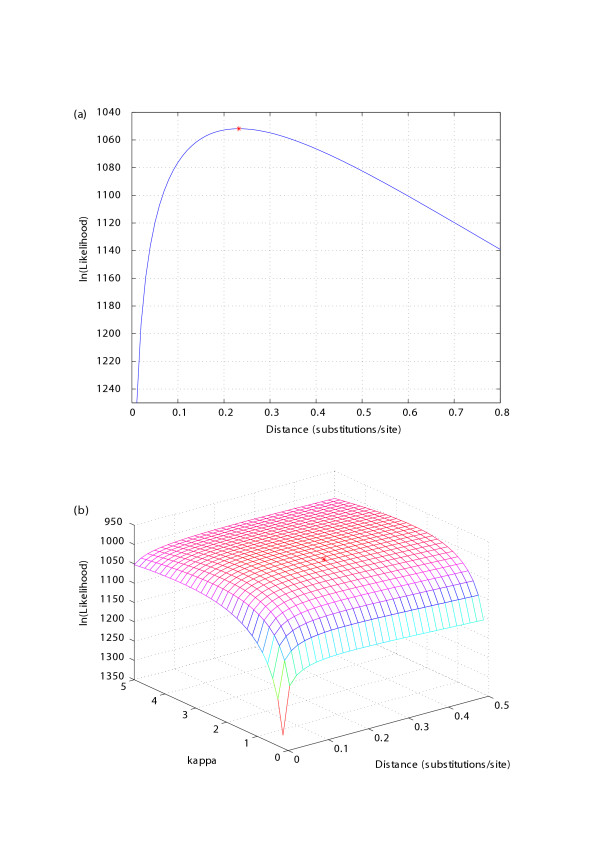
**Log-likelihood of evolutionary distance**. (a) Likelihood as function of K2P distance. The distance is estimated by maximising the likelihood of the alignment with the bias of transitions to transversions, *kappa*, held fixed. (b) Likelihood as a function of distance and *kappa*. Both the distance and *kappa *are optimised simultaneously. The maximum likelihood peaks are marked with *. The two sequences used are the coding regions of Tamarin eosinophil-derived neurotoxin (Acc. No.: U24099) and human eosinophil cationic gene (Acc. No: NM_002935).

In most cases we wish to estimate *t *instead of calculating *L *as a function of *t*, so the function optimlikelidist (model, s1, s2) will search for the *t *that maximises the likelihood by using the Nelder-Mead simplex (direct search) method, while holding the other parameters in the model at fixed values. This constraint can be relaxed by allowing every parameter in the model to be estimated by functions, such as optimlikelidistk2p, that can estimate both *t *and the model's parameters. Figure (1a and 1b) illustrates the estimation of the evolutionary distance between two ribonuclease genes through the fixed- and free-parameter K2P models, respectively. When the K2P model's parameter, *kappa*, is fixed, the result and trace of the optimisation process is illustrated by the graph of *L *and *t *(Fig. 1a). When *kappa *is a free parameter, a surface shows the result and trace of the optimisation process (Fig. 1b).

When calculating the likelihood of a phylogenetic tree, where *s*1 and *s*2 are two (descendant) nodes in a tree joined to an internal (ancestor) node, *s*_*a*_, we must sum over all possible assignments of nucleotides to *s*_*a *_to get the likelihood of the distance between *s*1 and *s*2. Consequently, the number of possible combinations of nucleotides becomes too large to be enumerated for even moderately sized trees. The *pruning algorithm *introduced by Felsenstein [19] takes advantage of the tree topology to evaluate the summation in a computationally efficient (but mathematically equivalent) manner. This and a simple and elegant mapping from a 'parentheses' encoding of a tree to the matrix equation for calculating the likelihood of a tree, developed in the MATLAB software, PHYLLAB [20], have been adopted in likelitree.

### Combination of functions

Basic operations can be combined to give more complicated functions. A simple combination of the function to extract the fourfold degenerate sites with the function to calculate GC content produces a new function (countgc4) that determines the GC content at 4-fold degenerate sites (GC4). A subfunction for calculating synonymous and nonsynonymous differences between two codons, getsynnonsyndiff, can be converted into a program for calculating codon volatility [21] with trivial effort. Similarly, karlinsig which returns Karlin's genomic signature (the dinucleotide relative abundance or bias) for a given sequence can be easily re-formulated to estimate relative *di-codon *frequencies, which may be a new index of biological signals in a coding sequence. In addition, the menu-driven user interface, MBEGUI, is also a good example illustrating the power of combination of basic MBEToolbox functions.

### Graphics and GUI

**Figure 2:**
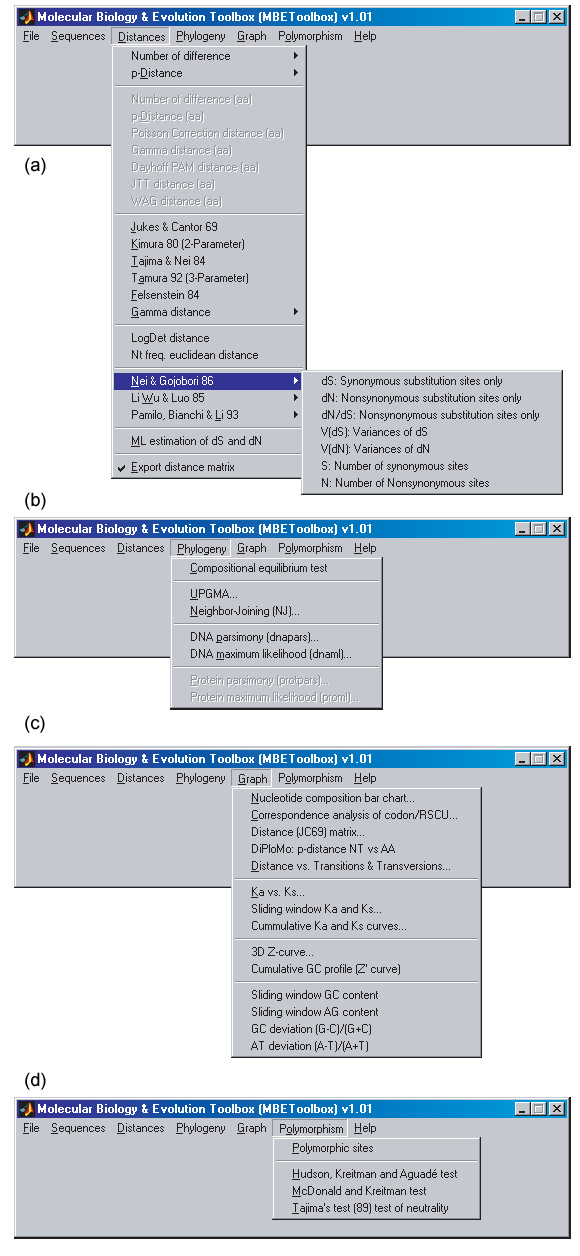
**MBEToolbox GUI**. (a) Distances submenu; (b) Phylogeny submenu; (c) Graph submenu; and (d) Polymorphism submenu.

Good visualisation is essential for successful numerical model building. Leveraging the rich graphics functionality of MATLAB, MBEToolbox provides a number of functions that can be used to create graphic output, such as scatterplots of *K*_*s *_vs *K*_*a*_, plots of the number of transitions and transversions against genetic distance, sliding window analyses on a nucleotide sequence and the Z-curve (a 3-dimensional curve representation of a DNA sequence [22]). A simple menu-driven graphical user interface (GUI) has been developed by using GUIDE in MATLAB. The top menu includes File, Sequences, Distances, Phylogeny, Graph, Polymorphism and Help submenus (Fig. 2). It aids the usage of the most frequently required functions so that users do not have to run any scripts or functions from the MATLAB command line in most cases.

## Results and discussion

### Vectorization simplifies programming

MATLAB is a matrix language, which means it is designed for vector and matrix operations. Programming can be simplified and made more efficient by using algorithms that take advantage of *vectorization *(converting for and while loops to the equivalent vector or matrix operations). The MATLAB compiler in version 7.0 will automatically recognize and vectorize loops without recursion. An example of vectorization is the calculation of Z-scores [23] for Smith-Waterman alignments [24] to give a measure of the significance of an alignment score against a background of scores from randomly generated sequences with the same composition and length. Hence, Z-scores are designed to overcome the bias due to the composition of the alignment and are usually calculated by comparing an actual alignment score with the scores obtained on a set of random sequences generated by a Monte-Carlo process. The Z-score is defined as:

*Z*(*A*, *B*) = (*S*(*A*, *B*) - *mean*)/*standard deviation*

where *S*(*A*, *B*) is the Smith-Waterman (S-W) score between two sequences *A *and *B*. The mean and standard deviation are taken from realignments of the permuted sequences. The algorithm is implemented as follows in MATLAB with as few as 15 lines of code:

function [z,z_raw] = zscores(s1,s2,nboot)

ml = length(s1);

m2 = length(s2);

% Initialise two vectors holding Z-score of

% s1_rep and s2_rep, i.e., replicate samples

% of sequences s1 and s2.

v_z1 = zeros(1,nboot);

v_z2 = zeros(1,nboot);

z_raw = smithwaterman(s1,s2);

for (k = 1:nboot),

   s1_rep = s1(:,randperm(m1));

   v_z1(1,k) = smithwaterman(s1_rep, s2) ;

   s2_rep = s2(:,randperm(m2));

   v_z2(1,k) = smithwaterman(s1, s2_rep);

end

z1 = (z_raw-mean(v_z1))./std(v_z1);

z2 = (z_raw-mean(v_z2))./std(v_z2);

z = min(z1,z2);

where randperm(n) is a vector function returning a random permutation of the integers from 1 to *n *and smithwaterman performs local alignment by the standard dynamic programming technique.

### Extensibility

An important distinction between compiled languages with subroutine libraries and interactive environments like MATLAB is the ease with which problems can be specified and solved in the latter. Moreover, MATLAB toolboxes are traditionally organised in a less object-oriented mode and, consequently, functions are more independent of each other and easier to combine and extend. Several examples were given in the Implementation section.

### Comparison with other toolboxes

Some other toolboxes have been developed in MATLAB for bioinformatics related analyses. These include PHYLLAB [20] and MATARRAY [25] as well as the bioinformatics toolbox developed by MATHWORKS. Other examples can be found at the link and file exchange maintained at MATLAB CENTRAL [26]. PHYLLAB is a molecular phylogeny toolbox which also provides some functions for sequence and tree input and manipulation. Its main focus is on creating a maximum likelihood tree based on Bayesian principles using a Markov chain Monte Carlo method to compute posterior parameter distributions. MATARRAY is focussed on the analysis of gene expression data from microarrays and provides normalization and clustering functions but does not address molecular evolution. The bioinformatics toolbox from MATHWORKS provides a range of bioinformatics functions, including some related to molecular evolution.

MBEToolbox provides a much broader range of molecular evolution related functions and phylogenetic methods than either the more specialized PHYLLAB project or the more general bioinformatics toolbox from MATHWORKS. These extra functions include IO in PHYLIP format, statistical and sequence manipulation functions relevant to molecular evolution (e.g. count segregating sites), evolutionary distance calculation for nucleic and amino acid sequences, phylogeny inference functions and graphic plots relevant to molecular evolution (e.g. *K*_*a *_vs *K*_*s*_). As such it makes an important contribution to the bioinformatics analyses that can be performed in the MATLAB environment.

### A novel enhanced window analysis

To test for the selective pressures in the different lineages of a phylogenetic tree, the nonsynonymous to synonymous rate ratio (*K*_*a*_/*K*_*s*_) is normally estimated [27-29]. Values of *K*_*a*_/*K*_*s *_= 1, > 1, or < 1 indicate neutrality, positive selection, or purifying selection, respectively. However, *K*_*s *_and *K*_*a *_are measurements of average synonymous and nonsynonymous substitutions per site along the whole length of the sequences. Average *K*_*s *_and *K*_*a*_values give neither the pattern of intragenic fluctuation of selective constraints, nor region- or site-specific information. A sliding window method is usually adopted to examine the *intragenic *pattern of the substitution rates and to test for the occurrence of significant clusters of variant regions [30-33]. Significant heterogeneity in *K*_*s *_would indicate that the neutral substitution rate varies across the gene, whereas heterogeneity in *K*_*a*_may indicate that selective constraints vary along the gene. The results and accuracy of sliding window methods, either overlapping or non-overlapping, depend on both the size of the window and the moving distance adopted. Large window lengths may obliterate the details of patterns in *K*_*s *_or *K*_*a*_, whereas small window lengths usually result in larger statistical fluctuations. Hence, the resolution of a sliding window is usually limited.

A mathematical formalism, similar to the Z'-curve [34], is introduced here to solve this problem. Consider a subsequence based analysis of *K*_*s *_or *K*_*a*_. In the *n*-th step, count the *cumulative *numbers of *K*_*s *_or *K*_*a *_occurring from the first to the *n*-th nucleotide position in the gene sequences being inspected. Let  denote either *K*_*s *_or *K*_*a *_and  denote the cumulative  at the *n*-th sequence position.  is usually an approximately mono-increasing linear function of *n*. The points (, *n*), *n *= 1, 2, ..., *N *are fit by a least square method to a linear function, *f*() = *βn*, to give a straight line with *β *being its slope. We define



The two-dimensional curve of ( ~ *n*) gives an alternative representation of the normal sliding window curve.

**Figure 3:**
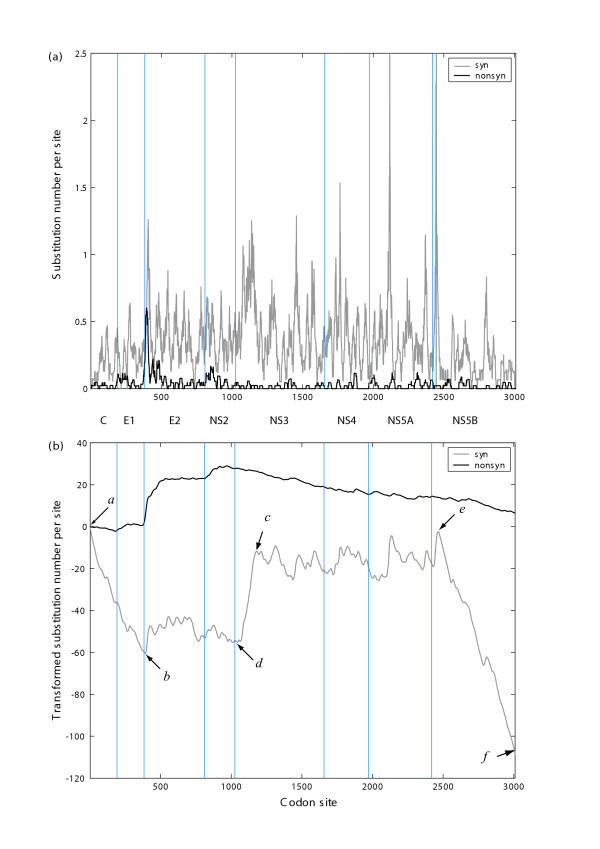
**A comparison between sliding window and enhanced sliding window methods**. Sliding window analysis of *K*_*s *_and *K*_*a *_for the concatenated coding regions of two hepatitis C virus strains, HCV-JS and HCV-JT. The number of codons for the C, El, E2, NS2, NS3, NS4, NS5A, and NS5B genes are 191, 192, 426, 217, 631, 315, 447, and 591, respectively. The different coding regions are separated by vertical lines. (a) illustrates the result of a normal sliding window analysis; (b) illustrates the result of the enhanced sliding window analysis. Beginnings and ends of regions poor in synonymous substitutions (*slope *< 0) are indicated by the arrows ***a ***and ***b ***(genes C and El) and ***e ***and ***f ***(gene NS5B). A region rich in synonymous substitutions (*slope *> 0) in gene NS3 is indicated by arrows *c *and ***d***.

To compare these two curve representations, the example dataset of Suzuki and Gojobori [35], which contains the coding regions of two hepatitis C virus strains (HCV-JS – Genbank Acc.: D85516 and HCV-JT – Genbank Acc.: D11168), was used. The entire coding sequence is divided into eight regions (C, El, E2, NS2, NS3, NS4, NS5A, NS5B). Some of the coding regions have been combined as these short ORFs are unlikely to yield meaningful *K*_*s *_and *K*_*a *_values. The reduction of *K*_*s *_in the C, El and NS5B regions, as well as its elevation in NS3, which have been shown in previous studies [35], are not clear in a standard sliding window representation (Fig. 3a). In contrast a sharp increase in the ( ~ *n*) curve (Fig. 3b), indicates an increase in , while a drop in the curve indicates a decrease in . This new method has been implemented in the function plotSlidingKaKs. Since it is derived from the sliding window method, it is called the enhanced sliding window method.

### Limitations

The current version of this toolbox lacks novel algorithms yet it implements a variety of existing algorithms. There are some limitations in the practical use of MBEToolbox. First, though the toolbox provides many methods to infer and handle sequence and evolutionary analyses, the full range of these features can only be accessed through the MATLAB command line interface, as in the majority of MATLAB packages. Second, some of the functions cannot handle ambiguous nucleotide or amino acid codes in the sequences. The future development of MBEToolbox will overcome these present limitations.

## Conclusion

The MBEToolbox project is an ongoing effort to provide an easy-to-use, yet powerful, analysis environment for molecular biology and evolution. Currently, it offers a substantial set of frequently used functions to manipulate sequences, to calculate genetic distances, to infer phylogenetic trees and related analyses. MBEToolbox is a useful tool which should inspire evolutionary biologists to take advantage of the MATLAB environment.

## Availability and requirements

**Project name: **MBEToolbox

**Project web page: **



**Operating system: **WINDOWS 95/98/2000/XP

**Programming language: **MATLAB 6.0 or higher

**Other requirements: **Statistics Toolbox

**License: **GPL

**Any restrictions on use by non-academics: **License needed

## Authors' contributions

JJC designed and implemented the software and wrote the draft of the manuscript. DKS participated in the design and revised the manuscript. XX participated in the design and provided suggestions for future development. KYY supervised and participated in the design of the study. All authors read and approved the final version of the manuscript.

## References

[B1] Felsenstein J (1989). PHYLIP – Phylogeny Inference Package (Version 3.2). Cladistics.

[B2] Thompson J, Higgins D, Gibson T (1994). CLUSTAL W: improving the sensitivity of progressive multiple sequence alignment through sequence weighting, position-specific gap penalties and weight matrix choice. Nucl Acids Res.

[B3] Jukes TH, Cantor C, Munro HN (1969). Evolution of protein molecules. Mammalian Protein Metabolism.

[B4] Kimura M (1980). A simple method for estimating evolutionary rates of base substitutions through comparative studies of nucleotide sequences. J Mol Evol.

[B5] Hasegawa M, Kishino H, Yano T (1985). Dating of the human-ape splitting by a molecular clock of mitochondrial DNA. J Mol Evol.

[B6] Nei M, Kumar S (2000). Molecular evolution and phylogenetics.

[B7] Lanave C, Preparata G, Saccone C, Serio G (1984). A new method for calculating evolutionary substitution rates. J Mol Evol.

[B8] Tavare S (1986). Some probabilistic and statistical problems in the analysis of DNA sequences. Lectures on Mathematics in the Life Sciences.

[B9] Rodriguez F, Oliver JL, Marin A, Medina JR (1990). The general stochastic model of nucleotide substitution. J Theor Biol.

[B10] Yang Z (1994). Estimating the pattern of nucleotide substitution. J Mol Evol.

[B11] Steel MA (1994). Recovering a tree from the leaf colourations it generates under a Markov model. Appl Math Lett.

[B12] Lake JA (1994). Reconstructing evolutionary trees from DNA and protein sequences: paralinear distances. Proc Natl Acad Sci USA.

[B13] Nei M, Gojobori T (1986). Simple methods for estimating the numbers of synonymous and nonsynonymous nucleotide substitutions. Mol Biol Evol.

[B14] Li WH, Wu CI, Luo CC (1985). A new method for estimating synonymous and nonsynonymous rates of nucleotide substitution considering the relative likelihood of nucleotide and codon changes. Mol Biol Evol.

[B15] Li WH (1993). Unbiased estimation of the rates of synonymous and nonsynonymous substitution. J Mol Evol.

[B16] Pamilo P, Bianchi NO (1993). Evolution of the Zfx and Zfy genes: rates and interdependence between the genes. Mol Biol Evol.

[B17] Yang Z (2000). Phylogenetic Analysis by Maximum Likelihood (PAML) Version 30.

[B18] Saitou N, Nei M (1987). The neighbor-joining method: a new method for reconstructing phylogenetic trees. Mol Biol Evol.

[B19] Felsenstein J (1981). Evolutionary trees from DNA sequences: a maximum likelihood approach. J Mol Evol.

[B20] Rzhetsky A, Morozov P (2001). Markov chain Monte Carlo computation of confidence intervals for substitution-rate variation in proteins. Pac Symp Biocomput.

[B21] Plotkin JB, Dushoff J, Fraser HB (2004). Detecting selection using a single genome sequence of M. tuberculosis and P. falciparum. Nature.

[B22] Zhang R, Zhang CT (1994). Z curves, an intutive tool for visualizing and analyzing the DNA sequences. J Biomol Struct Dyn.

[B23] Pearson WR, Lipman DJ (1988). Improved tools for biological sequence comparison. Proc Natil Acad Sci U S A.

[B24] Smith TF, Waterman MS (1981). Identification of common molecular subsequences. J Mol Biol.

[B25] Venet D (2003). MatArray: a Matlab toolbox for microarray data. Bioinformatics.

[B26] MATLAB Central. http://www.mathworks.com/matlabcentral/.

[B27] Sharp PM (1997). In search of molecular darwinism. Nature.

[B28] Akashi H (1999). Within- and between-species DNA sequence variation and the 'footprint' of natural selection. Gene.

[B29] Crandall K, Kelsey C, Imamichi H, Lane H, Salzman N (1999). Parallel evolution of drug resistance in HIV: failure of nonsynonymous/synonymous substitution rate ratio to detect selection. Mol Biol Evol.

[B30] Clark AG, Kao T (1991). Nonsynonymous Substitution at Shared Polymorphic Sites Among Self-Incompatibility Alleles of Solanaceae. Proc Natl Acad Sci USA.

[B31] Ina Y (1994). ODEN: a program package for molecular evolutionary analysis and database search of DNA and amino acid sequences. Comput Appl Biosci.

[B32] Endo T, Ikeo K, Gojobori T (1996). Large-scale search for genes on which positive selection may operate. Mol Biol Evol.

[B33] Choi SS, Lahn BT (2003). Adaptive evolution of MRG, a neuron-specific gene family implicated in nociception. Genome Res.

[B34] Zhang CT, Wang J, Zhang R (2001). A novel method to calculate the G+C content of genomic DNA sequences. J Biomol Struct Dyn.

[B35] Suzuki Y, Gojobori T, Salemi M, Vandamme A (2003). Analysis of coding sequences. The phylogenetic handbook: a practical approach to DNA and protein phylogeny.

